# The miR-221-5p/RAD18/RAD51 Axis Regulates DNA Damage Tolerance and Homologous Recombination to Drive Platinum Resistance in Ovarian Cancer

**DOI:** 10.21203/rs.3.rs-11040918/v1

**Published:** 2026-09-29

**Authors:** Tasmin Rahman Omy, Naresh Sah, Subash Kairamkonda, Chinnadurai Mani, Md Ariful Islam, Mark B. Reedy, Kenneth P. Nephew, Komaraiah Palle

**Affiliations:** Texas Tech University Health Sciences Center; Texas Tech University Health Sciences Center; Texas Tech University Health Sciences Center; Texas Tech University Health Sciences Center; Texas Tech University Health Sciences Center; Texas Tech University Health Sciences Center; Indiana University School of Medicine-Bloomington; Texas Tech University Health Sciences Center

**Keywords:** miR-221-5p, RAD18, RAD51, chemoresistance, platinum resistance, HR deficiency, BRCAness, PARP inhibitor

## Abstract

Platinum resistance remains a major barrier in epithelial ovarian cancer (EOC) treatment, while upregulation of the DNA damage response (DDR) is a hallmark of chemoresistance, and the underlying epigenetic mechanisms driving this adaptation remain poorly understood. Here, we identify a novel post-transcriptional regulatory axis involving miR-221-5p that governs two critical DDR effectors: RAD18, which mediates DNA damage tolerance through translesion synthesis (TLS), and RAD51, the central recombinase for homologous recombination (HR). Although the miR-221/222 cluster has been traditionally categorized as oncogenic, we demonstrated that the miR-221-5p arm functions as a potent tumor suppressor in EOC. Bioinformatics and luciferase reporter assays confirmed that miR-221-5p directly targets the 3′UTRs of both *RAD18* and *RAD51*. In EOC clinical specimens and cell lines, miR-221-5p downregulation inversely correlated with RAD18/RAD51 expression. Functionally, miR-221-5p restoration suppressed platinum-induced PCNA mono-ubiquitination and HR, inducing a “functional BRCAness” that sensitized both established and patient-derived primary EOC cells to carboplatin and PARP inhibition. Furthermore, in xenograft models, stable miR-221-5p expression significantly reduced the tumor burden. Collectively, our results reveal a previously unrecognized miR-221-5p/RAD18/RAD51 axis that may contribute to therapeutic responses in EOC and provide a rationale for therapeutic strategies targeting RAD18/RAD51-driven DNA repair pathways.

## Introduction

More than 320,000 women are diagnosed with ovarian cancer each year globally, and over 207,000 die from the disease [1][2]. Epithelial ovarian cancer (EOC) remains the most lethal gynecological malignancy worldwide[3]; it is characterized by insidious progression and is often diagnosed at an advanced stage. Although radical debulking surgery followed by platinum-taxane chemotherapy initially achieves high response rates, approximately 70% of patients succumb to disease recurrence[4]. This clinical relapse is almost universally driven by acquired chemoresistance, which represents the most formidable barrier to long-term survival. Projections for 2025 underscore this persistent burden, with over 20,000 new cases and 12,000 deaths estimated in the U.S. alone [5].

The development of chemoresistance is a multifactorial process, yet upregulation of the DNA damage response (DDR) is a hallmark strategy for tumor cell survival[6][8]. By efficiently repairing or bypassing the DNA crosslinks and double-strand breaks induced by platinum agents, cancer cells maintain genomic instability and evade apoptosis[9][10]. While genetic alterations are primary drivers of DDR dysregulation, emerging evidence highlights epigenetic reprogramming, specifically the loss of regulatory microRNAs (miRNAs), as a critical post-transcriptional mechanism driving DDR adaptation[11] [12]. Among these miRNAs, the miR-221/222 cluster is widely recognized for its oncogenic potential[13] [14][15]. However, most research has focused on the 3p arm, leaving the specific functional role of the miR-221-5p strand in EOC largely unexplored[16].

Previously, we identified conserved miR-221-5p binding sites within the 3′ untranslated regions (3′-UTRs) of both *RAD18* and *RAD51*[17][18]. These proteins constitute a dual-arm survival mechanism. RAD18 orchestrates DNA damage tolerance (DDT) via translesion synthesis (TLS), allowing cells to replicate past bulky platinum adducts[19][20]. RAD51 is the central recombinase for high-fidelity homologous recombination (HR) repair of DSBs[21][22]. In the present study, we tested the hypothesis that the downregulation or loss of miR-221-5p serves as a derepressing mechanism for the RAD18/RAD51 axis, thereby driving the increased expression of these repair proteins to promote DNA damage adaptation in EOC cells. We further hypothesized that by driving a coordinated network that shields the cancer genome from diverse therapeutic insults, simultaneous upregulation of these factors by repression of miR-221-5p contributes to platinum-resistant EOC.

Here, we identified miR-221-5p as a novel regulator of the DDR effectors RAD18 and RAD51, two functionally distinct proteins that play key roles in DNA repair. By using a combination of luciferase reporter assays, 3D organoid cultures, and *in vivo* xenograft models, we demonstrated that restoring miR-221-5p effectively collapses this repair axis, inducing a state of “functional BRCAness”. This restoration not only induces homologous recombination deficiency (HRD) and increases persistent DNA strand breaks, but also significantly resensitizes resistant EOC cells to carboplatin and the PARP inhibitor olaparib. Collectively, our findings delineate a novel miR-221-5p/RAD18/RAD51 epigenetic regulatory axis and suggest that restoration of miR-221-5p expression may represent a precision oncology strategy to overcome the formidable challenge of chemoresistance in aggressive EOC.

## Materials and methods

### Cell lines, culture conditions and reagents

The human EOC cell lines OVCAR3, OVCAR4, OVCAR8, OV90, ES2, A2780, and SKOV3 were purchased from the American Type Culture Collection (ATCC; Manassas, VA). To model acquired chemoresistance, isogenic platinum-sensitive/resistant pairs (A2780 vs. A2780/CP70 and SKOV3 vs. SKOV3/CP20) were utilized as previously described [23][24][25]. Immortalized human fallopian tube epithelial cells (FTECs) served as nonmalignant controls [26].

All the cell lines were cultured in their respective vendor-recommended media (e.g., DMEM, RPMI-1640, or DMEM/F12) supplemented with 10% fetal bovine serum (FBS; Omega Scientific Inc.) and 1% penicillin‒streptomycin (Corning). Cultures were maintained in a humidified atmosphere at 37°C with 5% CO_2_. All lines were routinely screened for *Mycoplasma* contamination via PCR-based detection (Abcam). Carboplatin (Carbo; Selleckchem) and Olaparib (Ola; Thermo Scientific Chemicals) were dissolved in ultrapure water and DMSO respectively to create stock solutions and applied at the concentrations and durations specified in the figure legends.

### Bioinformatics and Clinical Correlation Analysis

#### Clinical survival analysis

The prognostic value of *RAD18* and *RAD51* mRNA expression was evaluated via the Kaplan‒Meier (KM) plotter database [27]. We analyzed a combined cohort of 614 ovarian cancer patients derived from The Cancer Genome Atlas (TCGA) and GSE14764 datasets. Patients were stratified into high- or low-expression cohorts using the median transcript level as the discriminatory cutoff. Overall survival (OS) and progression-free survival (PFS) were compared via the log-rank test. Survival outcomes are reported as hazard ratios (HRs) with 95% confidence intervals (CIs) and corresponding *P* values.

#### Differential miRNA expression analysis

To identify clinically relevant miRNAs, we reanalyzed the Gene Expression Omnibus (GEO) dataset GSE235525[28] and compared the miRNA expression profiles from the serum of patients with high-grade serous ovarian cancer (HGSOC) with those of healthy controls. Differentially expressed miRNAs (DEMs) were identified via the GEO2R interactive tool. Statistical significance was defined by an adjusted *P* < 0.05 and a log_2_-fold change > 1.

#### *In silico* gene expression validation

The differential expression of *RAD18* and *RAD51* between malignant ovarian tissues and normal controls was validated via the Gene Expression Profiling Interactive Analysis (GEPIA) portal[29]. This analysis integrated transcriptomic data from TCGA (tumor) and the Genotype-Tissue Expression (GTEx) project (normal) via a standardized processing pipeline.

### Dual-luciferase 3’UTR reporter assay

To confirm the direct post-transcriptional regulation of *RAD18* and *RAD51* by miR-221-5p, individual GoClone 3′ UTR reporter constructs (Active Motif, Carlsbad, CA) were utilized. These vectors contain the firefly luciferase open reading frame (ORF) fused to the 3′ UTR regions of *RAD18* or *RAD51* harboring the predicted miR-221-5p binding sites.

ES2 and OVCAR8 cells were seeded in 96-well plates and co-transfected with 100 ng of the respective luciferase reporter vectors and 50 nM of either the miR-221-5p mimics (Cat# C-301163-01-0020) or nontargeting control mimics (Cat# CN-001000-01-20; Horizon Discovery) via Lipofectamine RNAiMAX (Life Technologies). After 48 h of incubation, luciferase activity was quantified via the LightSwitch Assay System (SwitchGear Genomics) on a Tecan microplate reader according to the manufacturer’s instructions. To account for variations in transfection efficiency, firefly luciferase activity was normalized to either a co-transfected constitutive reporter or to the total protein concentration (as per the LightSwitch system protocol). The results are expressed as the relative luciferase activity compared with that of the nontargeting control.

### DNA Repair and Functional Assays

#### HR Reporter Assay:

To quantify HR efficiency, a functional DR-GFP reporter system was utilized as previously described [30]. The reporter consists of two nonfunctional GFP derivatives: a SceGFP gene containing an I-SceI recognition site and an internal stop codon and a truncated iGFP fragment. The pDR-GFP and pCBASceI plasmids were kindly provided by Dr. Maria Jasin (Addgene Plasmids #26477 and #26475).

OVCAR8 cells stably harboring the pDR-GFP cassette were transfected with the pCBASceI expression vector to induce a site-specific DNA double-strand break (DSB). The cells were simultaneously transfected with the miR-control or miR-221-5p mimics. Successful HR-mediated repair of the DSB via the iGFP fragment as a template restores the functional GFP open reading frame. At 48 h post-transfection, the percentage of GFP-positive cells was quantified via flow cytometry (BD Accuri) to determine the relative HR efficiency.

#### Alkaline comet assay

To assess global DNA damage and the persistence of strand breaks, an alkaline comet assay (single-cell gel electrophoresis) was performed via the OxiSelect 96-well comet assay kit (Cell Biolabs, San Diego, CA). Briefly, cells were treated with 100 μM carboplatin for 24 h, harvested, and embedded in 1% lowmelting-point agarose on specialized glass slides.

To permit the detection of both single- and double-strand breaks, the cells were subjected to alkaline lysis followed by DNA unwinding in alkaline electrophoresis buffer (pH > 13). Electrophoresis was conducted at 1 V/cm for 30 min to allow damaged DNA to migrate from the nucleus, forming a “comet tail.” Following neutralization and ethanol dehydration, the DNA was stained with Vista Green DNA Dye. Comets were visualized via a Zeiss Axio fluorescence microscope, and genomic damage was quantified by measuring the tail moment and tail DNA percentage in at least 50 randomly selected cells per condition via ImageJ software (NIH) equipped with the Open Comet plugin.

#### Clonogenic survival assay

Cellular viability and proliferative capacity following treatment were assessed via a clonogenic assay. The cells were seeded at low density (500–5,000 cells/well) in 6-well plates and allowed to adhere overnight. The cells were subsequently treated with the indicated concentrations of carboplatin or olaparib, following transfection with the miR-control or miR-221-5p mimics alone and in combination of carboplatin, and the cells were cultured for 7–10 days to allow for colony formation. Once the control wells reached the optimal density, the colonies were fixed with methanol and stained with 1% crystal violet solution.

Colonies consisting of ≥ 50 cells were manually or semiautomatically quantified via ImageJ software (NIH, Bethesda, MD). The surviving fraction was calculated by normalizing the plating efficiency of the treated groups to that of the respective nontargeting control (miR-NC) or vehicle-treated groups.

#### 3D culture and invasion assays

##### Patient-derived 3D Organoid Cultures

In this study, we used two epithelial primary ovarian cancer patient-derived cell lines (OV-TX-186 and OV-TX-285), obtained from the Texas Tech University Health Sciences Center (TTUHSC) Cancer Center Biorepository, to generate patient derived organoids. OV-TX-285 was derived from the ascitic fluid of a patient with progressive disease (PD) diagnosed with metastatic adenosquamous carcinoma of the ovary. OV-TX-186 was established from a secondary tumor in a right-sided mammary lymph node of a patient with metastatic high-grade serous ovarian carcinoma and progressive disease [31][32]. To promote anchorage-independent growth, cells were seeded in ultralow attachment (ULA) 6-well plates (Corning). Organoids were maintained in DMEM/F12 supplemented with 20% FBS, 1X B-27, 1X N-2, 20 ng/mL epidermal growth factor (EGF), and 20 ng/mL fibroblast growth factor (FGF). Organoid formation and morphology were monitored over a period of 7–14 days. Quantitative analysis of organoid diameter was performed using Nikon NIS-Elements (ND2) software.

##### 3D Matrix-Dependent Invasion Assay

To evaluate metastatic potential, multicellular spheroids were first generated in low-adhesion roundbottom 96-well plates at a density of 3,000 cells/well. Following a 72-h incubation to allow compact spheroid formation, 50 μL of Cultrex 3D Spheroid Invasion Matrix (R&D Systems) was overlaid. The invasive capacity of the spheroids was monitored over 3–6 days via a Nikon Eclipse TE confocal microscope. The degree of invasion was determined by measuring the invasive area and perimeter expansion relative to the day 0 core area via ImageJ software (NIH).

### Molecular analysis

#### Western Blotting Analysis

Total cellular proteins were extracted via RIPA lysis buffer (Thermo Fisher Scientific) supplemented with a protease and phosphatase inhibitor cocktail (Roche, Basel, Switzerland). Protein concentrations were determined via the Bradford assay (Bio-Rad). Equal amounts of protein were denatured and resolved via SDS‒PAGE via 8–12% gradient gels.

The resolved proteins were then electro-transferred onto nitrocellulose membranes (BioRad turbo transfer blot). The membranes were blocked for 1 h at room temperature with 5% nonfat dry milk in Tris-buffered saline containing 0.1% Tween-20 (TBST) to minimize nonspecific binding. The membranes were subsequently incubated overnight at 4°C with the indicated primary antibodies.

Following three washes with TBST, the membranes were incubated with horseradish peroxidase (HRP)-conjugated secondary antibodies for 1 h at room temperature. The protein bands were visualized via enhanced chemiluminescence (ECL) reagents (PerkinElmer) and captured via an Azure C300 imaging system (Azure Biosystems) or X-ray film developer. Densitometric quantification was performed via ImageJ software, with target protein levels normalized to vinculin, β-actin, or GAPDH as internal loading controls.

Quantitative real-time PCR (qRT‒PCR): Total RNA was isolated via a Quick-RNA MiniPrep kit (Zymo Research, Irvine, CA) according to the manufacturer’s instructions. The RNA concentration and purity were assessed via a NanoDrop spectrophotometer. For mRNA quantification, cDNA was synthesized from total RNA via a high-capacity cDNA reverse transcription kit (Invitrogen) with random hexamer primers. For miRNA quantification, cDNA was generated via the Mir-X miRNA First-Strand Synthesis Kit (TaKaRa Bio, Mountain View, CA), which utilizes a poly-A tailing strategy.

Quantitative PCR was performed with PowerUp SYBR Green Master Mix (Applied Biosystems) on a QuantStudio 12K Flex Real-Time PCR System. The specificity of the amplification was confirmed by melt curve analysis. Relative gene expression was calculated via the 2 − ΔΔCt method. The target mRNA levels were normalized to those of *GAPDH*, while the miRNA levels were normalized to those of the small nuclear RNA *U6*.

#### Cell cycle distribution analysis

Cell cycle progression was evaluated via flow cytometry after propidium iodide (PI) staining. Following transfection with the miR-control or miR-221-5p mimics alone and in combination of carboplatin at indicated dose, the cells were harvested via trypsinization, washed twice with ice-cold PBS, and fixed in 100% ice-cold ethanol at −20°C overnight.

Fixed cells were subsequently washed with PBS to remove residual ethanol and resuspended in a staining solution containing 50 μg/mL PI (Invitrogen) and 100 μg/mL RNase A (Thermo Scientific) to ensure DNA-specific binding. After a 30-minute incubation in the dark at room temperature, the DNA content was analyzed via a BD Accuri C6 flow cytometer (BD Biosciences). A minimum of 10,000 events were captured per sample. The percentages of cells in the G1, S and G2/M phases were quantified via ModFit LT 5.0 software (Verity Software House).

### Immunofluorescence and confocal microscopy

#### 2D Monolayer Imaging:

Cells were seeded into FluoroDishes (World Precision Instruments) and allowed to adhere overnight. Following treatment, the cells were fixed with 4% paraformaldehyde for 10 min at room temperature and permeabilized with 0.2% Triton X-100 in PBS for 3 min. To minimize nonspecific binding, the cells were blocked with 10% normal goat serum for 40 min. The samples were incubated overnight at 4°C with primary antibodies against 53BP1 (1:500; Cell Signaling, Cat# 4937) or RAD51 (1:200; Millipore, Cat# 65653). Following PBS washes, the cells were incubated with Alexa Fluor-conjugated secondary antibodies (1:500; Molecular Probes) for 2 h at room temperature. Nuclei were counterstained and mounted using Vectashield with DAPI (Vector Laboratories).

#### 3D Spheroid and Organoid Imaging:

For 3D cultures, spheroids were fixed in 4% PFA for 1 h, followed by extended permeabilization with 0.2% Triton X-100 for 20 min. After blocking, spheres were incubated overnight with anti-RAD18 (1:200; Cell Signaling, Cat# 9040) or RAD51 (1:200; Millipore, Cat# 65653) antibodies. To visualize the 3D architecture and cytoskeleton, spheres were stained with fluorescent phalloidin (Invitrogen, Cat# A12379) for 1 h.

All images were captured via a Nikon Eclipse TE confocal microscope equipped with a high-resolution Z-axis motor. For 3D analysis, Z-stacks were acquired at 5.0 μm intervals and processed as maximum intensity projections. Focus formation (defined as ≥ 5 foci per nucleus) and integrated fluorescence intensity were quantified via ImageJ software (NIH).

#### Animal studies and in vivo xenograft model

##### Lentiviral Vector Transduction and Stable Cell Line Generation

To generate stable miR-221-5p expression, SKOV3-Luc cells (expressing firefly luciferase) were seeded at a density of 5 × 10^4^ cells/cm^2^. After 24 h, the cells were transduced with lentiviral vectors encoding either miR-221-5p (pLenti-III-miR-GFP) or a nontargeting control (pLenti-III-miR-GFP-Blank; Applied Biological Materials, Richmond, BC, Canada) at a multiplicity of infection (MOI) of 15. The viral mixture was replaced with complete growth medium 24 h after transduction. The transduction efficiency was validated by monitoring GFP expression via fluorescence microscopy and confirmed by Western blot analysis of target repair proteins prior to implantation.

##### Intraperitoneal (i.p.) Xenograft Model

All animal experiments were performed in accordance with the NIH Guide for the Care and Use of Laboratory Animals and ARRIVE guidelines under protocols approved by the TTUHSC Institutional Animal Care and Use Committee (IACUC). Female athymic nude mice (8 weeks old) were purchased from The Jackson Laboratory and acclimated for one week prior to experimentation. After a week, mice were randomized into two groups (n = 6 per group). The mice received an intraperitoneal (i.p.) injection of 2×10^6^ SKOV3-Luc cells (control vs. miR-221-5p). Tumor progression and peritoneal dissemination were monitored 2–3 times weekly via bioluminescence imaging (IVIS; PerkinElmer). The mice were injected i.p. with D-luciferin (150 mg/kg) and imaged under isoflurane anesthesia. Mice were initially anesthetized with isoflurane (2–3%) in an induction chamber and subsequently transferred to the imaging manifold, where anesthesia was maintained via a nose cone (1.5–2% isoflurane). The bioluminescence intensity was quantified as the total radiance (photons·s^−1^·cm^−2^·sr^−1^). At the experimental endpoint (28–32 days post-injection), the mice were euthanized via carbon dioxide (CO_2_) inhalation followed by cervical dislocation as a secondary method to ensure death. The tumors were harvested, weighed, and fixed in 4% paraformaldehyde for subsequent immunohistochemistry (IHC) analysis.

### Statistical analysis

Statistical analyses were performed via GraphPad Prism v.8.4.3 (GraphPad Software, San Diego, CA). The quantitative data are presented as the means ± standard deviations (SDs) from at least three independent biological replicates, unless otherwise specified. For comparisons between two experimental groups, a two-tailed unpaired Student’s *t* test was employed, assuming equal variance. For experiments involving multiple groups or treatment conditions, one-way or two-way analysis of variance (ANOVA) followed by Tukey’s or Sidak’s post-hoc test for multiple comparisons was used. Longitudinal tumor growth data from IVIS imaging were analyzed via two-way repeated-measures ANOVA. A *P* value < 0.05 was considered statistically significant. The significance levels are denoted in the figures as follows: *P < 0.05, **P < 0.01, ***P < 0.001, ****P < 0.0001.

## Results

### RAD18 and RAD51 Overexpression is Correlated with Poor Prognosis and miR-221-5p Downregulation in Ovarian Cancer

To investigate the clinical relevance of DNA damage response (DDR) dysregulation in EOC, we first investigated the prognostic significance of two central DDR effectors: RAD51, the primary recombinase facilitating homologous recombination (HR), and RAD18, the E3 ubiquitin ligase critical for translesion synthesis (TLS). Analysis of The Cancer Genome Atlas (TCGA) datasets via GEPIA [29] and KMplotter [27] showed that high mRNA expression of both *RAD51* (Fig. 1A) and *RAD18* (Fig. 1B) was significantly correlated with reduced overall survival (OS) and progression-free survival (PFS) in EOC patients (Fig. 1C, 1D). Validation at the protein level across a panel of EOC cell lines confirmed a significant upregulation of RAD51 and RAD18 compared with those in nonmalignant FTEC cells (Fig. 1E, 1F).

On the basis of our previous findings regarding miRNA-mediated DDR regulation[12], we performed a reanalysis of the GEO dataset (GSE235525)[28]. This revealed that miR-221-5p was one of the most significantly downregulated miRNAs in clinical EOC samples compared with normal tissue (Supplemental Fig. 1A, 1B and Supplemental Table 1). To further characterize this regulatory network between *miR-221-5p* and *RAD51* and *RAD18*, we analyzed their expression profiles in a panel of established EOC cell lines and nonmalignant FTEC controls. Quantitative RT‒PCR (qRT‒PCR) across our cell line panel established an inverse correlation. *miR-221-5p* was nearly undetectable in malignant cell lines in which *RAD18* and *RAD51* were overexpressed (Fig. 1G). Functionally, ectopic restoration of miR-221-5p in OVCAR8 cells significantly suppressed the malignant phenotype, as evidenced by a significant reduction in clonogenic survival and 3D spheroid growth (Fig. 1H–1J). Similar results were observed in ES2 (Supplemental Fig. 1C, 1D) and SKOV3 cells (Supplemental Fig. 1E-1H). Importantly, flow cytometric analysis indicated that these effects were not due to general arrest of the cell cycle, as the G1/S/G2 distributions remained stable (Supplemental Fig. 2A-2D), suggesting that miR-221-5p acts primarily by impairing proliferative signaling and antitumorigenic effects.

### miR-221-5p targets the 3′UTRs ofRAD18andRAD51and suppresses their expression

We next investigated whether the observed inverse correlation between miR-221-5p and *RAD18* and *RAD51* expression was mediated by post-transcriptional silencing. Transfection of OVCAR8, ES2, and SKOV3 cells with miR-221-5p mimics resulted in a significant reduction in both RAD18 and RAD51 protein levels (Fig. 2A–2F). Consistent with these findings, *RAD18* and *RAD51* mRNA levels were also significantly decreased in OVCAR8 and ES2 cells following expression of miR-221-5p mimics (Supplemental Fig. 3A, 3B), indicating that miR-221-5p promotes target mRNA degradation in addition to translational repression.

To further validate this regulatory axis, we performed reciprocal rescue experiments using a miR-221-5p-specific inhibitor (anti-miR). While the miR-221-5p mimic significantly suppressed RAD18 and RAD51 protein expression in OVCAR8 cells, co-transfection with the corresponding anti-miR restored protein levels to near-baseline values (Fig. 2G–2I). These results demonstrate that repression of RAD18 and RAD51 is specifically mediated by miR-221-5p.

To determine whether RAD18 and RAD51 are targets of miR-221-5p, we first analyzed their 3’ untranslated regions (3′UTRs) using the TargetScan *in silico* prediction algorithm[17]. This analysis identified highly conserved miR-221-5p seed-matching sequences within the 3′UTRs of *RAD18* and *RAD51* (Fig. 2J). To experimentally validate these predicted interactions, luciferase reporter constructs containing the respective *RAD18* and *RAD51* 3′UTR regions harboring the predicated miR-221-5p binding sites (nucleotides 1522–1528 and 1702–1708, respectively) were generated (Supplemental Fig. 3C). Dual-luciferase reporter assays revealed that ectopic miR-221-5p mimic expression significantly reduced luciferase activity in both OVCAR8 and ES2 cells compared with that in cells transfected with a nontargeting control mimic (Fig. 2K). Collectively, these findings support a model in which miR-221-5p negatively regulates *RAD18* and *RAD51* through their 3’UTR-dependent mechanisms, resulting in decreased mRNA and protein expression and suppression of the RAD18/RAD51 DNA repair axis.

Box plots summarizing the mRNA levels of *RAD51* (A) and *RAD18* (B) in the TCGA ovarian serous cystadenocarcinoma cohort. Statistical significance was determined by a two-tailed Student’s t test.

Kaplan–Meier survival curves for progression-free survival (PFS) and overall survival (OS) in patients stratified by high vs. low transcript expression of *RAD51* (C) or *RAD18* (D). Values were calculated via the log-rank test.

Western blot analysis (E) and corresponding densitometric quantification (F) of RAD51 and RAD18 in nonmalignant fallopian tube epithelial cells (FTECs) and a panel of ovarian cancer cell lines. The data represent the means ± SDs of ≥ 3 independent experiments (*P < 0.05, **P < 0.01, ***P < 0.001; two-tailed *t*-test).

(G) Relative expression of *RAD18*, *RAD51*, and *miR-221-5p* in FTEC and EOC cell lines as determined by qRT‒PCR. The mRNA and miRNA levels were normalized to those of *GAPDH* and *U6*, respectively, and plotted as percentages of FTEC expression. (The values represent the means ± SDs from ≥ 3 independent experiments (*P* < 0.05, *P < 0.01, **P < 0.001; two-tailed *t*-test).

(H) Representative images of low-density colony assays and survival fraction of OVCAR8 cells following transfection with control miRNA or miR-221-5p mimics. Bars represent the mean ± SD from three biological replicates (**P < 0.01; two-tailed *t*-test).

Representation of 3D spheroid cultures of OVCAR8 cells following transfection with control miRNA or miR-221-5p mimics and violin plot of spheroid diameters measured from the 3D assay. Each point represents an individual spheroid (n ≥ 100 spheroids per group, diameter larger than 50 μm). The data are presented as the means ± SD (*P* < 0.05, *P < 0.01, **P < 0.001; two-tailed *t*-test).

### miR-221-5p suppresses growth, stemness and invasion in patient-derived primary EOC models

To evaluate the clinical relevance of the miR-221-5p/RAD18/RAD51 regulatory axis, we extended our studies to two patient-derived primary EOC cell line models (OV-TX-186 and OV-TX-285). Consistent with observations in established ovarian cancer cell lines, ectopic expression of miR-221-5p significantly reduced RAD18 and RAD51 protein levels in both the primary cultures (Supplemental Fig. 4).

To determine the functional consequences of miR-221-5p restoration on tumor-initiating properties, we performed 3D organoid formation assays. Ectopic expression of miR-221-5p mimics significantly impaired organoid-forming efficiency and growth in both OV-TX-186 and OV-TX-285 cells (Fig. 3A–3D), indicating a reduction in self-renewal capacity and stemness-associated properties. Consistent with these findings, immunofluorescence analysis of OV-TX-186-derived organoids revealed robust downregulation of RAD18 and RAD51 throughout organoid structure following miR-221-5p restoration (Fig. 3E–3F).

Western blot analyses of RAD18 and RAD51 in OVCAR8 (A, B), ES2 (C, D) and SKOV3 (E, F) cells 48 h after transfection with a control miRNA or a miR-221-5p mimic. The corresponding bar graphs in B, D and F show densitometric quantification of protein expression normalized to vinculin (mean ± SD, n = 3 independent replicates; ****P < 0.0001, two-tailed *t* test).

Functional rescue experiment in OVCAR8 cells. The cells were transfected with the control miR, the miR-221-5p mimic or the miR-221-5p inhibitor (anti-miR). Immunoblotting (G) and quantification (H, I) demonstrated that the miR-221-5p-mediated downregulation of RAD18 and RAD51 was effectively reversed by the inhibitor, confirming the specificity of this regulatory axis.

(J) Schematic representation of firefly luciferase reporter constructs containing the 3′-untranslatedregions (3′ UTRs) of *RAD51* or *RAD18*. Conserved miR-221-5p binding sequences within each 3′ UTR are highlighted.

(K) Relative luciferase activity in OVCAR8 and ES2 cells co-transfected with miR-221-5p mimics and theindicated 3′ UTR reporter vectors. The values were normalized to those of internal controls. The data are presented as the means ± SDs of n = 3; *P < 0.05, **P < 0.01, by two-tailed t test.

Given the established role of stem-like tumor cells in metastatic progression, we next assessed the effect of miR-221-5p on the invasive capacity of primary EOC cells using 3D Matrigel invasion assays. Compared with control-miR transfected cells, miR-221-5p mimic expression significantly reduced invasive outgrowth in both the primary models (Fig. 3G–3I), demonstrating that miR-221-5p restrains aggressive cellular behaviors in a physiologically relevant extracellular matrix environment.

Collectively, these data demonstrate that restoration of miR-221-5p suppresses growth, stemness-associated phenotypes, and invasive potential in patient derived EOC models, corresponding with reduced RAD18/RAD51 expression. These results further support a tumor-suppressive role for miR-221-5p and implicate the miR-221-5p/RAD18/RAD51 regulatory axis in the maintenance of aggressive ovarian cancer phenotypes.

### miR-221-5p overexpression attenuates ovarian tumor growth in an intraperitoneal xenograft model

To evaluate the potential *in vivo* effects of miR-221-5p expression, SKOV3-Luc cells were stably transduced with lentiviral particles encoding either a miR-221-5p mimic or a nontargeting control. Stable integration and knockdown of the candidate target proteins RAD18 and RAD51 were confirmed via western blotting before implantation (Supplemental Fig. 5A). Stable miR-221-5p expression was also associated with decreased in *in vitro* 3D spheroid growth, supporting persistence of the phenotype observed with the lentiviral construct (Supplemental Fig. 5B–5C).

Representative brightfield images (A, C) and quantitative size distribution violin plots (B, D) of 3D organoids derived from primary patient-derived EOC models OV-TX-186 (A, B) and OV-TX-285 (C, D) post-transfection with control miRNA or miR-221-5p mimics. Each point in the violin plots represents an individual organoid (n ≥ 100 per condition with a size greater than 50 μm; **P < 0.01, ****P < 0.0001; two-tailed *t* test).

Representative 3D confocal immunofluorescence images of OV-TX-186 organoids stained for RAD18 (E) or RAD51 (F) (red) following miR-221-5p restoration. Nuclei were counterstained with DAPI (blue). Scale bars, 100 μm.

(G) Representative images of the 3D Matrigel-dependent invasion assay. OV-TX-186 organoids were embedded in a basement membrane matrix and monitored for invasive outward growth on days 0, 4, and 8.

(H) High-magnification confocal images of day 8 organoids from (G) stained with fluorescent phalloidin (green) to visualize cytoskeletal reorganization and actin cytoskeletal organization associated with invasion.

Quantification of the relative invasion area (%) over an 8-day period (G). The scale bar represents 100 μm (n = 3 per group; mean ± SD; ***P < 0.001; two-tailed *t* test).

Following molecular characterization, cells were implanted intraperitoneally (IP) into nude mice (n = 6 per group) to establish an ovarian cancer xenograft model. Tumor progression was monitored noninvasively by bioluminescence imaging (IVIS). To allow for stable engraftment, longitudinal bioluminescence measurements were initiated 10 days after implantation (Fig. 4A). Compared with the control cohort, mice with miR-221-5p expressing cells showed a progressive reduction in bioluminescence signal intensity, consistent with lower tumor burden over time (Fig. 4B–4D). Two-way ANOVA incorporating treatment group and time demonstrated a significant difference in overall tumor burden between the groups.

To correlate these physiological outcomes with our proposed molecular mechanism, harvested tumor tissues were analyzed via immunohistochemistry (IHC). Consistent with the *in vitro* findings, miR-221-5p was associated with lower RAD51 protein levels within the tumor parenchyma (Fig. 4E, 4G). Staining for the proliferation marker Ki67 also indicated reduced proliferative activity in miR-221-5p-expressing xenografts compared with control xenografts (Fig. 4E,4F). Together, these findings suggest that miR-221-5p expression may suppress ovarian tumor growth *in vivo*, at least in part, through reduced RAD51 and RAD18 expression and decreased cellular proliferation.

(A) Schematic of the experimental workflow. Athymic nude mice (n = 6/group) were IP injected withSKOV3-Luc cells stably expressing either a control miRNA or miR-221-5p. Tumor progression was monitored weekly starting on day 10 post implantation via bioluminescence imaging (IVIS).

(B) Quantification of total radiance (photons s^−1^ cm^−2^ sr^−1^) measured at the day 28 endpoint. The data represent the means ± SDs; n = 6 mice per group; *P < 0.05, two-tailed *t*-test).

(C) Representative IVIS bioluminescence images of mice from the control and miR-221-5p groups on day 28. The pseudo color scale indicates the radiance intensity, reflecting the tumor burden.

(D) Longitudinal tumor growth curves based on bioluminescence radiance over time. Statistical significance was determined by two-way repeated-measures ANOVA (mean ± SD; **P < 0.01).

(E) Representative immunohistochemistry (IHC) images of harvested tumor sections stained for the proliferation marker Ki67 (top) and the DNA repair protein RAD51 (bottom). Scale bars, 100 μm in the main images and 50 μm in the zoomed insets.

(F, G) Quantitative analysis of Ki67-positive (F) and RAD51-positive (G) cells, expressed as a percentage of total cells per high-power field (n = 3 tumors per group; data represent the mean ± SD; (****P < 0.0001, two-tailed *t*-test).

### miR-221-5p restoration is associated with reduced DNA damage tolerance and increased carboplatin sensitivity in EOC cells

To determine how miR-221-5p-mediated regulation of RAD18 and RAD51 influences DNA repair pathways, we examined downstream DDR signaling. A critical step in TLS is the RAD18-dependent mono-ubiquitination of PCNA (Ub-PCNA)[20][33], a modification that facilitates replication fork to bypass platinum-DNA adducts. Consistent with RAD18 downregulation, miR-221-5p restoration significantly reduced Ub-PCNA levels following carboplatin exposure across multiple EOC cell lines (Fig. 5A–C). We also observed a concomitant decrease in monoubiquitinated FANCD2(Ub-FANCD2), a central marker of the Fanconi anemia (FA) pathway[34][35], suggesting that miR-221-5p-mediated repression of RAD18 extends beyond TLS to disrupt multiple DNA damage tolerance and repair pathways [36][37][38][39][40].

We next evaluated impact of miR-221-5p expression on HR repair efficiency using DR-GFP reporter assays [41]in OVCAR8 cells. Ectopic expression of miR-221-5p significantly reduced the proportion of GFP-positive cells following I-SceI-induced DNA double strand breaks (DSBs) compared to control-miR transfected cells, indicating impaired HR repair efficiency (Fig. 5D). Consistent with this finding, alkaline comet assays showed significantly increased comet tail lengths in miR-221-5p transfected cells, reflecting the persistence of unrepaired DNA lesions (Fig. 5E–5F). Supporting these results, immunofluorescence microscopy demonstrated robust RAD51 repair foci formation in carboplatin treated control cells, whereas miR-221-5p-transfected cells exhibited significantly reduced RAD51 foci. This reduction was accompanied by a corresponding increase in 53BP1 foci, indicative of the accumulation of unrepaired DSBs (Fig. 6A–6D).

To determine whether these defects in DNA damage tolerance and repair translated into altered therapeutic responses, we performed colony formation assays in OVCAR8 and SKOV3 cells exposed to increased concentrations of carboplatin (up to 5 μM). Control-miR-transfected cells exhibited the expected dose-dependent decrease in clonogenic survival following carboplatin treatment. In contrast, restoration of miR-221-5p resulted in a significant greater reduction in colony-forming capacity across the tested drug concentrations (Fig. 6E–6H). To determine whether these effects were influenced by alterations in cell cycle progression, we performed cell cycle analysis by flow cytometry. No significant differences were observed in the proportion of cells in the G1, S, or G2/M phases between the control and miR-221-5p-transfected cells (Supplemental Fig. 2A-2D) in any treatment (carboplatin) conditions. Together, these data suggest that miR-221-5p promotes carboplatin sensitivity, at least in part, by impaired RAD18-mediated TLS and RAD51-mediated HR.

Western blot analysis of OVCAR8 (A), ES2 (B) and SKOV3 (C) cells transfected with a control miRNA or a miR-221-5p mimic and exposed to carboplatin (Carbo, 300 μM) for 24 h.

(D) OVCAR8 cells were transfected with pDR-GFP plasmid and selected with 5 μg/ml puromycin. Stably expressing cells were simultaneously transfected with miR-control or miR-221-5p mimics along with the pCBASceI expression vector. At 48 h post transfection, the number of GFP-positive cells was quantified via flow cytometry (BD Accuri) to determine the relative HR efficiency (mean ± SD of three independent experiments; ****P < 0.0001; two-tailed *t* test).

(E) Alkaline comet assay images of SKOV3 cells transfected with control miRNA or the miR-221-5p mimic and treated with carboplatin (100 μM) or vehicle for 24 h.

(F) Quantification of the comet tail area from (E). Each point represents an individual nucleus; horizontal bars denote the mean ± SD (more than 25 cells per condition). (***P < 0.001, ****P < 0.0001; two-tailed *t* test; ns, not significant).

### miR-221-5p restoration overcomes platinum resistance and sensitizes EOC cells to carboplatin and olaparib

To investigate whether the RAD18/RAD51 axis are associated with acquired platinum resistance, we evaluated two isogenic pairs of platinum-sensitive (A2780, SKOV3) and their acquired-resistant derivatives (A2780/CP70, SKOV3/CP20) EOC cell lines. Western blot analysis showed elevated RAD18 and RAD51 protein levels in the resistant SKOV3/CP20 and A2780/CP70 cells compared with their sensitive parental counterparts (Supplemental Fig. 6A-B). Increased expression of these repair proteins was accompanied by enhanced clonogenic survival following carboplatin treatment (Supplemental Fig. 6C-H).

Consistent with our proposed model, ectopic restoration of miR-221-5p in these resistant cells was associated with reduced RAD18 and RAD51 protein levels (Fig. 7A, 7D). Importantly, miR-221-5p restoration also resensitized resistant cells to carboplatin, resulting in a significant reduction in their colony forming ability compared with control-miR transfected resistant cells (Fig. 7B, 7C, 7E, 7F). Together, these results support a role for the miR-221-5p-RAD18-RAD51 axis in platinum resistance and suggest that restoration of miR-221-5p may represent a strategy to overcome treatment resistance in EOC.

Representative immunofluorescence images of OVCAR8 cells transfected with control miRNA or miR-221-5p mimics, followed by treatment with vehicle or carboplatin (100 μM, 24 h). The cells were stained for 53BP1 (A), a marker of double-strand breaks, or RAD51 (B), a marker of homologous recombination repair. Scale bar represents 10 μm.

(C) Quantitative analysis of the percentage of cells positive for 53BP1 foci (≥ 5 foci/nucleus). The datarepresent the means ± SEMs from more than 75 cells per condition (*P < 0.05, **P < 0.01, ***P < 0.001; two-tailed *t* test).

(D) Quantitative analysis of the percentage of cells positive for RAD51 foci (≥ 5 foci/nucleus). The data represent the means ± SEMs from more than 75 cells per condition. (****P < 0.0001; two-tailed *t* test).

Representative images of low-density colony formation assays in OVCAR8 (E) and SKOV3 (F) cells transfected with control or miR-221-5p mimics and treated with increasing doses of carboplatin (0–5 μM).

Clonogenic survival curves represent the survival fraction of OVCAR8 (G) and SKOV3 (H) cells from (E, F). The results revealed significant chemosensitization following miR-221-5p restoration. The data represent the means ± SEMs of three independent experiments (*P < 0.05, **P < 0.01 vs. control; two-tailed *t* tests).

Given the established role of RAD18 in promoting RAD51 recruitment to stalled or collapsed replication forks and facilitating HR repair[42][43], we next investigated whether miR-221-5p-mediated reduction of RAD18/RAD51 axis increased sensitivity to PARP inhibition. In OVCAR8, A2780, and SKOV3 cells, olaparib reduced clonogenic survival in a dose-dependent manner, notably, this effect was significantly enhanced in cells transfected with miR-221-5p mimics (Fig. 8A–8C and Supplemental Fig. 7A–7B). These findings suggest that restoration of miR-221-5p compromises HR- repair capacity and thereby sensitizes EOC to PARP inhibition.

To determine whether this effect extends to a more physiologically relevant model, we evaluated the combination of miR-221-5p restoration and olaparib combination in two primary patient-derived organoids (OV-TX-186 and OV-TX-285) models of EOC. Consistent with the findings in established cell lines, miR-221-5p restoration enhanced olaparib sensitivity in both organoid models, resulting in significantly reduced organoid growth and survival compared with control-treated organoids (Fig. 8D–G).

Collectively, these results support an association between elevated RAD18 and RAD51 expression and acquired platinum resistance in EOC. Restoration of miR-221-5p suppressed expression of both DNA repair factors, resensitized platinum-resistant cells to carboplatin, and enhanced sensitivity to olaparib in both established cell lines and patient-derived organoids. Together, these data suggest that miR-221-5p-mediated modulation of DNA repair pathways may represent a promising strategy for overcoming therapeutic resistance and enhancing therapeutic efficacy in EOC.

(A) Immunoblot analysis of RAD18 and RAD51 expression in platinum resistant SKOV3/CP20 cells 48 h after transfection with control miRNA or miR-221-5p mimics.

Representative images (B) and corresponding survival fraction curves (C) from clonogenic survival assays of SKOV3/CP20 cells treated with increasing concentrations of carboplatin (0–10 μM) following treatment with control miRNA or miR-221-5p mimics (Mean ± SD, n = 3, *P < 0.05, **P < 0.01 vs. control; two-tailed *t* tests).

(D) Immunoblots showing the depletion of RAD18 and RAD51 in platinum-resistant A2780/CP70 cells 48 h after transfection with a control miRNA or a miR-221-5p mimic.

Representative images (E) and quantitative survival fraction analysis (F) of a low-density colony formation assay in A2780/CP70 cells. Restoration of miR-221-5p significantly sensitized resistant cells to a carboplatin gradient (0–10 μM). The data are presented as the means ± SDs from three independent biological replicates (***P < 0.001, two-tailed t-test).

(A) Representative images of low-density clonogenic assays. OVCAR8 (left) and A2780 (right) cells were transfected with control miRNA or miR-221-5p mimics and treated with increasing concentrations of the PARP inhibitor olaparib (0–1.5 μM).

Survival dose‒response curves quantifying colony-forming efficiency (normalized to untreated controls) for OVCAR8 (B) and A2780 (C) cells. The data are presented as the means ± SD of three independent experiments. (*P < 0.05, two-tailed *t*-test).

Representative brightfield images (D) and corresponding violin plots (E) showing the distribution of patient-derived OV-TX-186 organoids size following miR-221-5p mimic transfection and olaparib treatment (0, 1.5, and 3 μM).

Representative brightfield images (F) and corresponding violin plots (G) showing the distribution of patient-derived OV-TX-285 organoids size following miR-221-5p mimic transfection and olaparib treatment (0, 1, and 1.5 μM).

For organoid analysis, approximately 50 organoids per condition were measured. Organoids 50 μm in diameter were included in the quantification. The data are presented as the means ± SDs of three biological replicates. Organoid sizes greater than 50 μm were counted (*P < 0.05, **P < 0.01; two-tailed *t*-test).

## Discussion

EOC remains one of the most lethal gynecological malignancy [44][45], in part because acquired platinum resistance commonly emerges after initially effective treatment [46][43]. Although surgical debulking and platinum-based chemotherapy can produce meaningful clinical responses, adaptive changes in DNA damage response (DDR) pathways may contribute to relapse and treatment failure. In this study, we investigated the miR-221-5p/RAD18/RAD51 axis as a potential contributor to DNA repair capacity and therapeutic response in EOC models[47]. Our findings support a model in which reduced miR-221-5p expression may relieve posttranscriptional repression of RAD18 and RAD51, thereby increasing activity in repair-associated pathways involving TLS and HR.

The clinical relevance of RAD51 and RAD18 in EOC is supported by our TCGA-based analysis and by prior reports linking DNA repair pathway activity with outcome [48][49][50]. RAD51 is a central recombinase in HR, and increased RAD51 expression or activity has been associated with earlier relapse and poorer survival in subsets of EOC patients [51][23]. RAD51 upregulation has also been described in high-grade serous ovarian cancer (HGSOC) and may contribute to restoration of HR competency, including contexts in which tumors evade platinum-induced DNA damage despite the absence of *BRCA1/2* reversion mutations[47][52].

RAD18 has similarly been implicated in DNA damage tolerance through its role in PCNA monoubiquitination, which facilitates TLS-mediated bypass of bulky platinum–DNA adducts [53] [54]. Our observation that RAD18 and RAD51 levels were higher in platinum-resistant isogenic pairs (A2780/CP70 and SKOV3/CP20) consistently with the possibility that resistant EOC cells engage more than one repair-associated mechanism. Rather than proving a coordinated dual-pathway program, these findings support further investigation into how TLS- and HR-associated factors cooperate during adaptation to platinum-induced genotoxic stress[55][56][57].

An important aspect of this work is the evaluation of miR-221-5p as a regulator of RAD18 and RAD51 expression. The miR-221/222 cluster has often been described as oncogenic, largely based on studies of the miR-221–3p arm and its repression of the p27Kip1 tumor suppressor [58][59]. Our data suggests that the 5p strand of miR-221 may have context-dependent tumor-suppressive effects in EOC models. This functional divergence between miRNA strands is consistent with the broader concept that mature strand selection and target specificity can produce distinct biological outcomes [60][61]. In our system, miR-221-5p reduced RAD18 and RAD51 expression and was associated with decreased Ub-PCNA and Ub-FANCD2 levels following carboplatin exposure. Together, these findings are consistent with a role for miR-221-5p in regulating DNA damage tolerance and HR-associated repair through these targets; however, further studies, including genetic rescue experiments, will be necessary to definitively establish the relative contribution of RAD18 and RAD51 to these effects.

Functionally, restoration of miR-221-5p was associated with reduced HR reporter activity, decreased RAD51 foci formation, increased 53BP1 foci, and greater DNA damage as measured by alkaline comet assay. Collectively, these observations are consistent with impaired repair capacity and persistence of DNA strand breaks after genotoxic stress. Importantly, flow cytometry showed no significant differences in cell cycle distribution with restoration of miR-221-5p in the presence or absence of carboplatin treatment, suggesting that the observed effects were not primarily attributable to alterations in cell cycle. Together, these results support the interpretation that miR-221-5p may contribute to carboplatin sensitivity by limiting RAD18- and RAD51-associated repair responses. However, further studies will be required to define the relative contribution of these pathways and determine whether additional miR-2215p targets contribute to these observed phenotypes.

From a translational perspective, the increased carboplatin and olaparib sensitivity observed after miR-221-5p restoration in cell lines and patient-derived organoids suggests potential therapeutic relevance, while also requiring cautious interpretation. PARP inhibitors (PARPis) are most effective in tumors with HRD; however, a substantial proportion of HGSOC tumors are HR-proficient and show limited responses to PARPis [47][62][63]. Our data raises the possibility that miR-221-5p restoration could be explored as a strategy to attenuate repair pathway activity and enhance sensitivity to platinum agents or PARP inhibition in selected ovarian cancer models. However, further validation in larger patient-derived cohorts, orthogonal miRNA delivery systems, and clinically relevant *in vivo* models will be necessary to assess the robustness, feasibility and therapeutic potential of this strategy before clinical implications can be inferred.

## Conclusion

Our study identifies a miR-221-5p-associated regulatory axis involving RAD18 and RAD51 in ovarian cancer models. The data supports a model in which reduced miR-221-5p expression represents one contributing factor to increased DNA damage tolerance and repair capacity, thereby promoting therapeutic resistance through derepression of RAD18 and RAD51. Conversely, restoration of miR-221-5p was associated with reduced TLS- and HR-associated signaling, increased persistence of DNA damage, and enhanced sensitivity to carboplatin and olaparib in the experimental systems tested. These findings provide a rationale for further investigation of miR-221-5p-based modulation of DNA repair pathways as a potential approach to improve therapeutic response in chemoresistant ovarian cancer.

### Limitations

While our findings identify miR-221-5p as a regulator of the RAD18/RAD51 axis in ovarian cancer models, several limitations should be considered. First, the study was conducted primarily in preclinical systems, including established cell lines, patient-derived organoids, and xenograft models. Validation in larger, clinically annotated patient cohorts will be necessary to establish the clinical relevance and prognostic significance of the miR-221-5p–RAD18–RAD51 axis.

Second, miRNAs are inherently pleiotropic and often regulate multiple targets in a context-dependent manner[64]. Therefore, although our data supports a role for RAD18 and RAD51 as functionally relevant targets of miR-221-5p, contributions from additional downstream effectors cannot be excluded.

Finally, although restoration of miR-221-5p demonstrated therapeutic potential in the experimental models evaluated, important translational challenges remain. The feasibility of miRNA-based therapy, including efficient and tumor-specific delivery, pharmacokinetic optimization, and long-term safety, will require further investigation before clinical application can be considered.

## Supplementary Material

Supplementary Files

This is a list of supplementary files associated with this preprint. Click to download.

- SupplementalFigures09142026.pdf

## Figures and Tables

**Figure 1:**
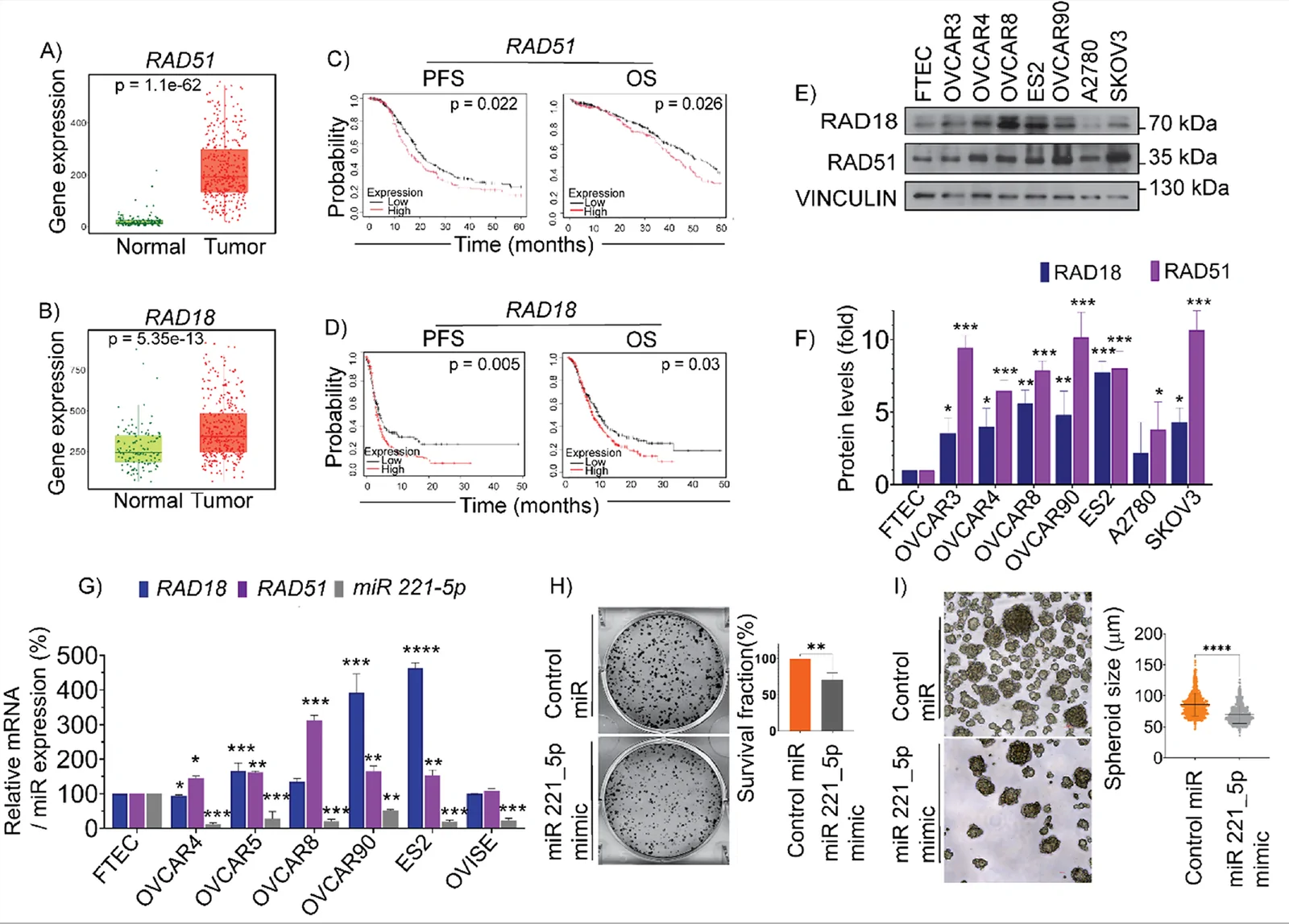
RAD18 and RAD51 overexpression is correlated with poor prognosis and is regulated by miR-221-5p in ovarian cancer.

**Figure 2:**
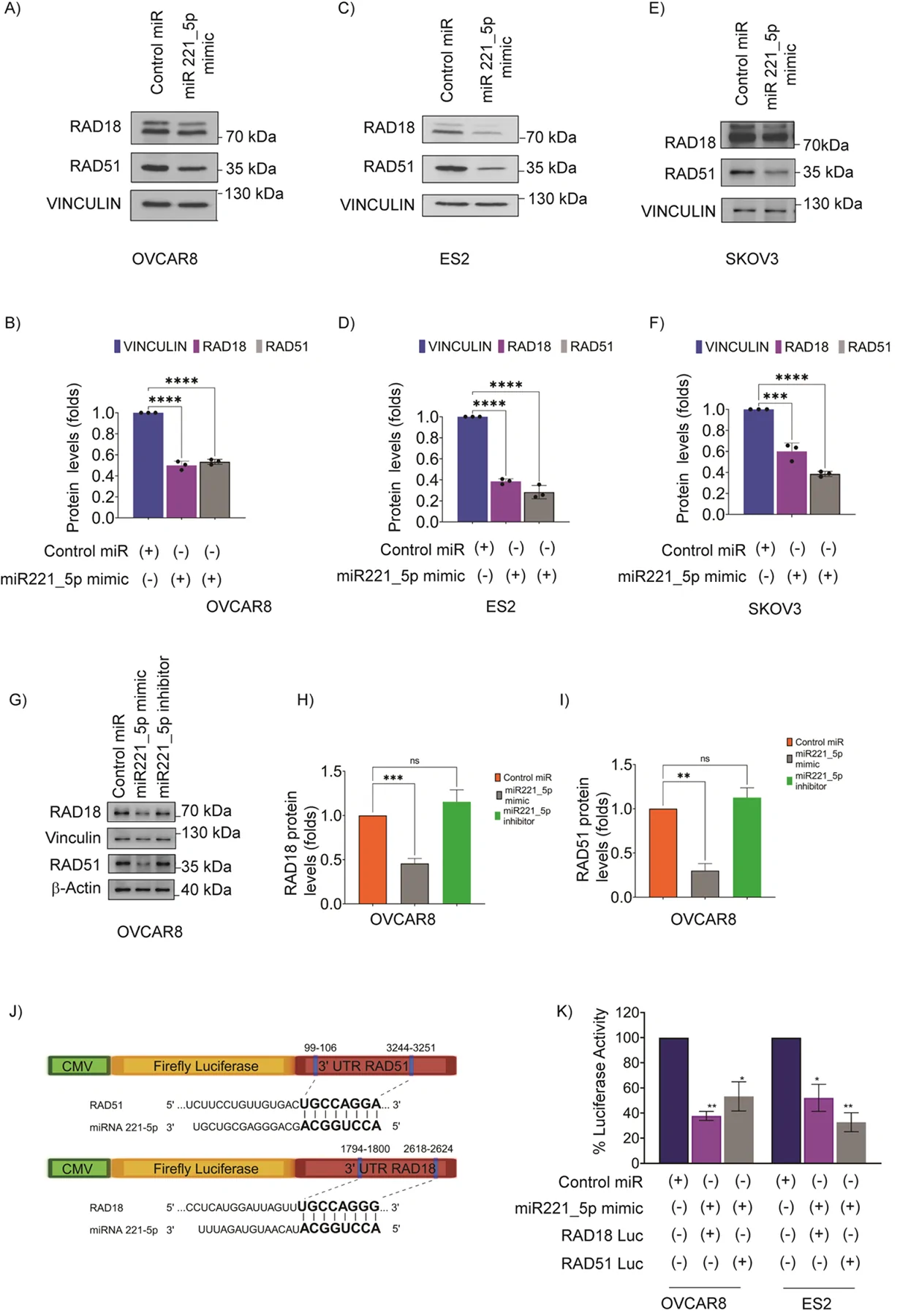
miR-221-5p directly suppresses RAD18 and RAD51 by targeting their 3′ UTRs.

**Figure 3:**
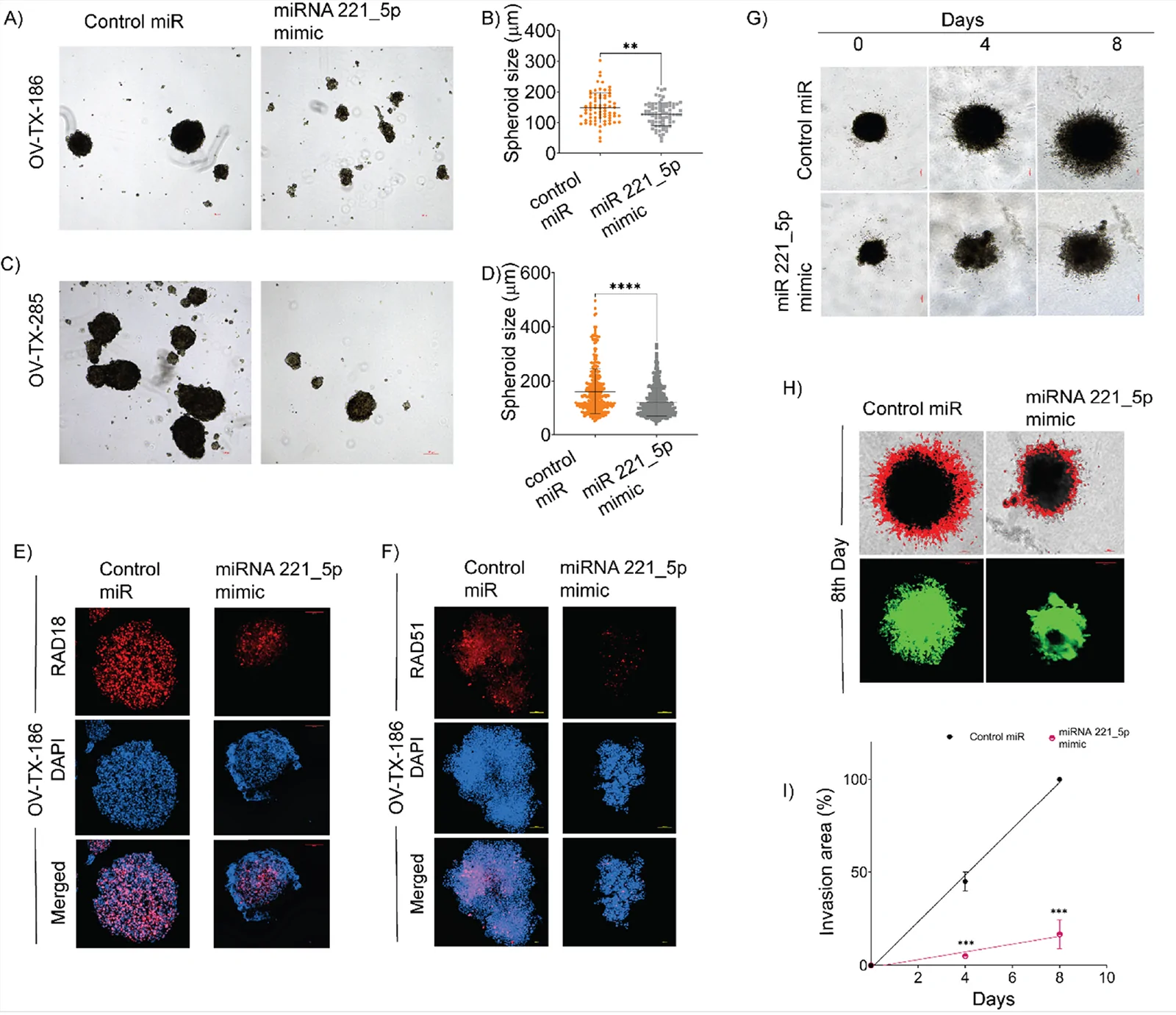
miR-221-5p attenuates the proliferative capacity and invasion potential ofprimary patient-derived EOC organoids.

**Figure 4:**
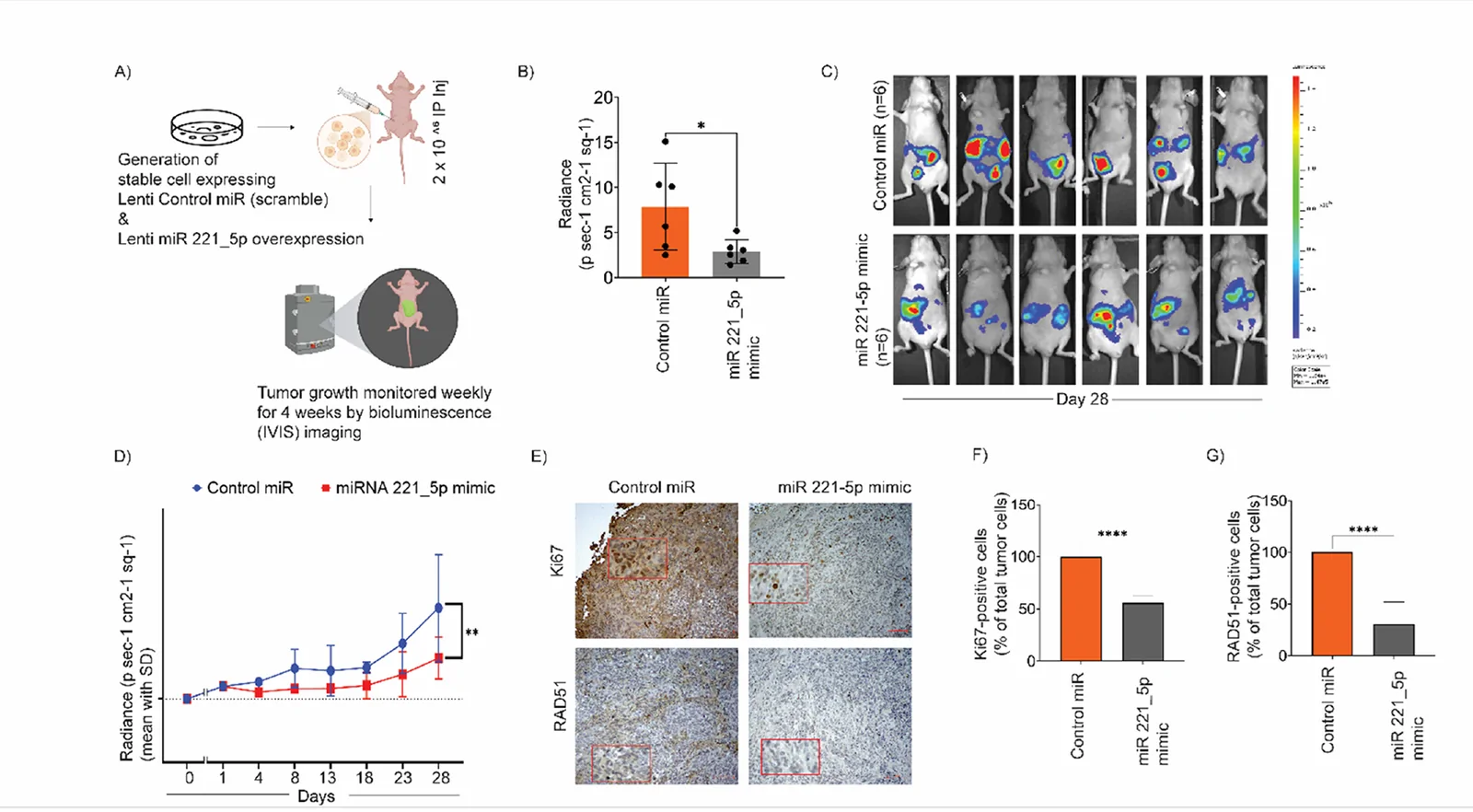
Stable miR-221-5p expression attenuates ovarian tumor growth and reduces RAD51 expression *in vivo*.

**Figure 5:**
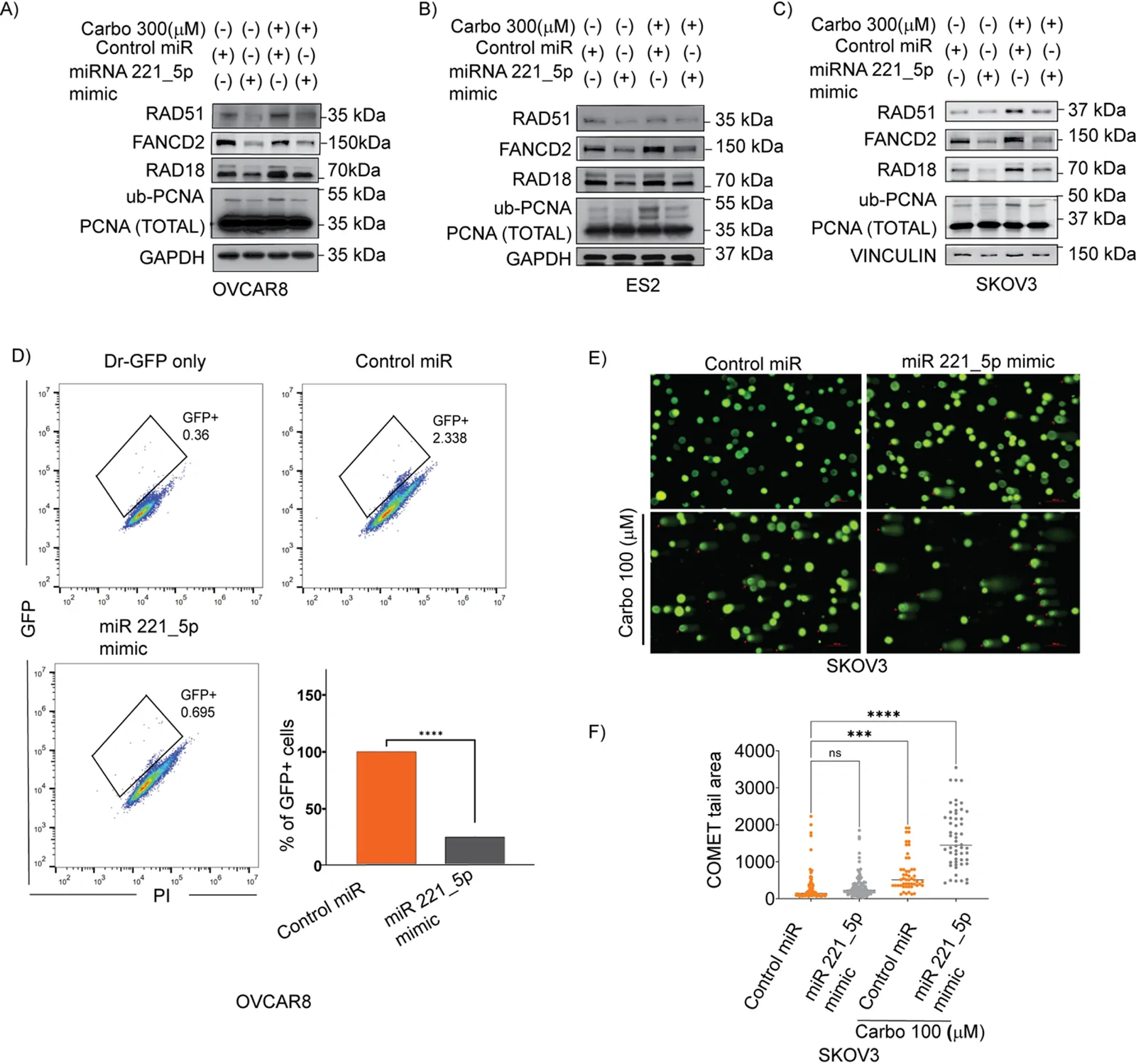
miR-221-5p restoration decreases DNA damage tolerance and homologous recombination, resensitizing EOC cells to carboplatin-induced damage.

**Figure 6:**
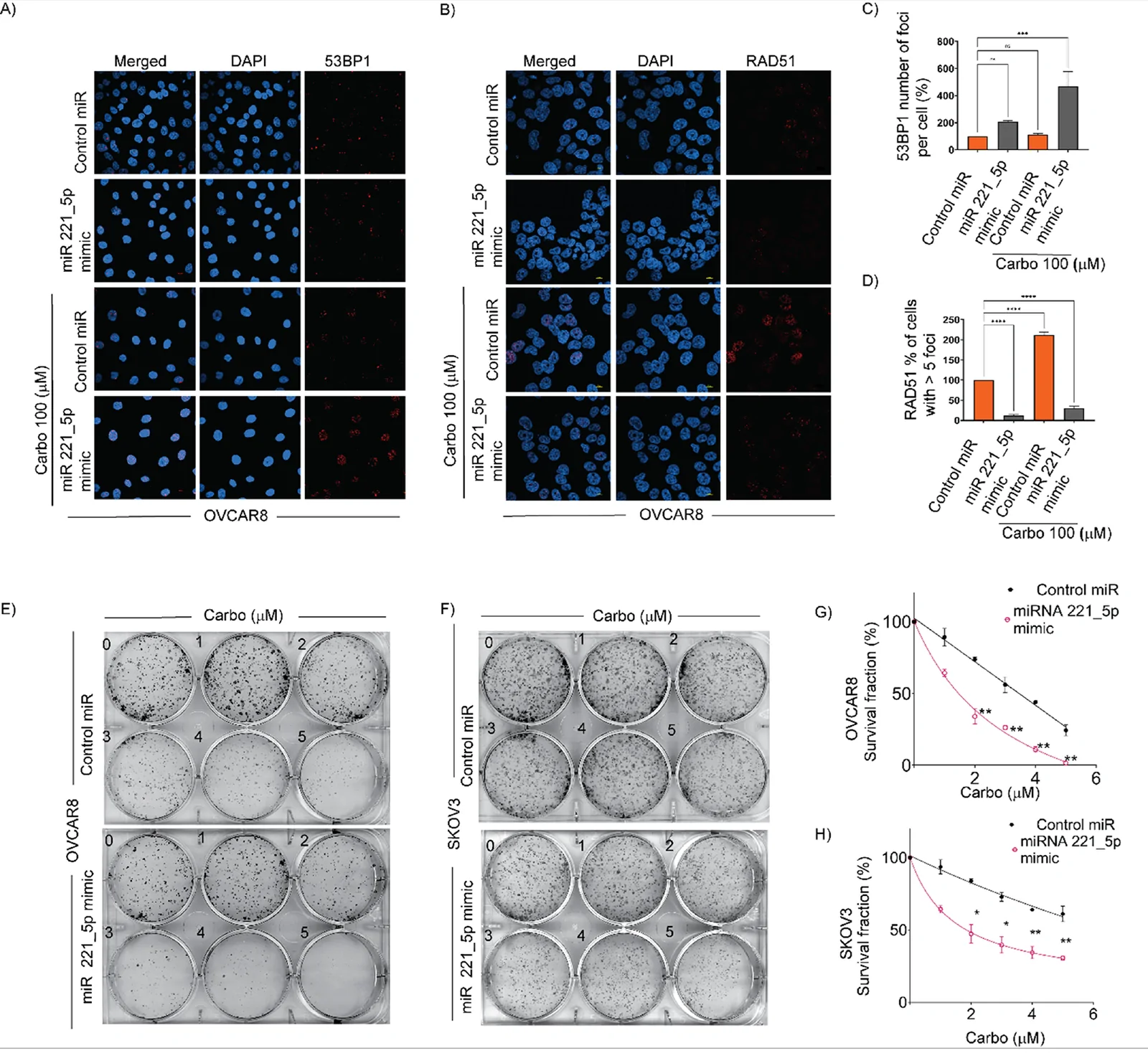
miR-221-5p disrupts RAD51 foci formation, promotes DNA damage accumulation, and sensitizes EOC cells to carboplatin.

**Figure 7:**
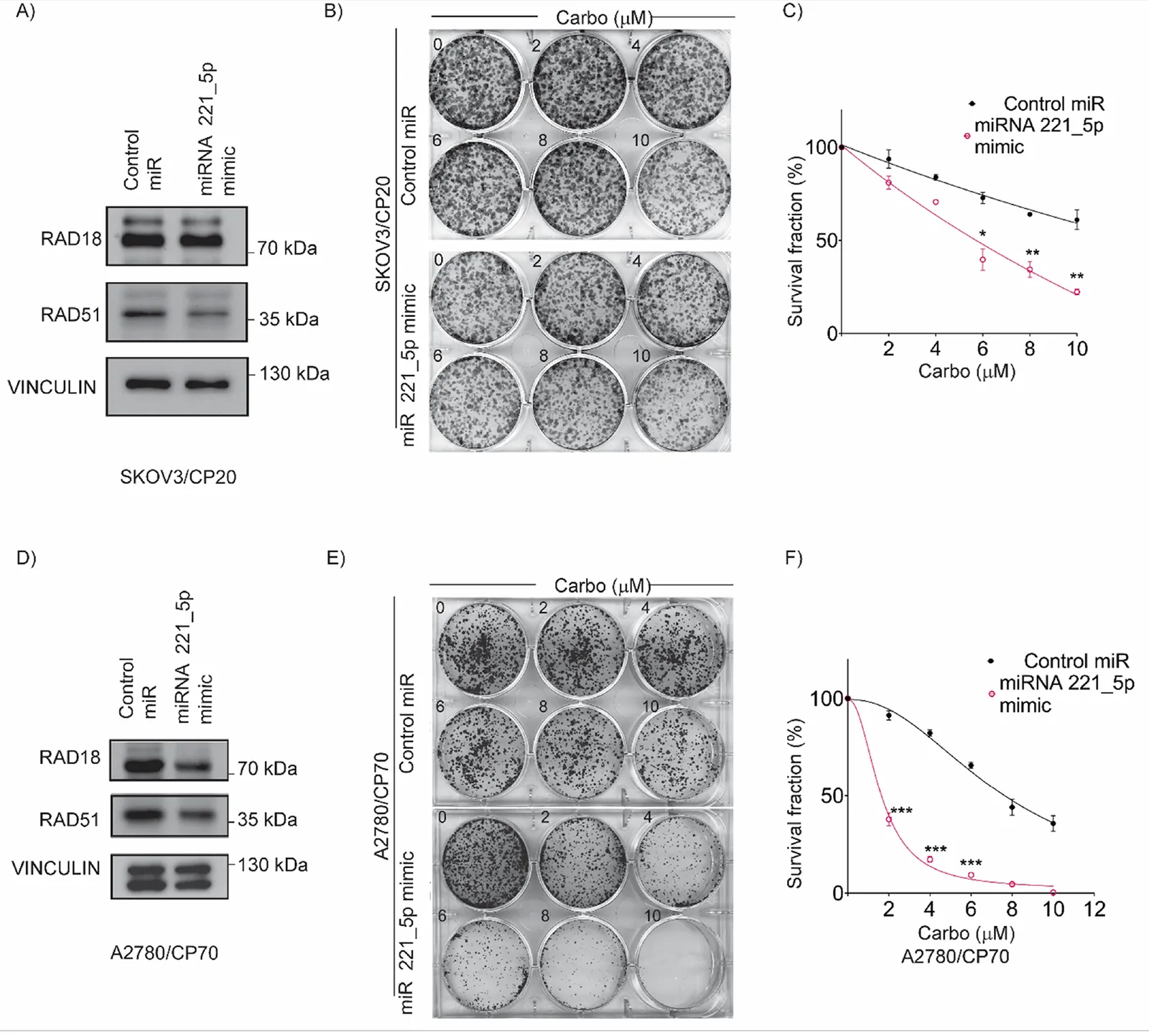
RAD18/RAD51 upregulation associated with platinum resistance, and miR-221-5p restoration resensitizes resistant cells.

**Figure 8:**
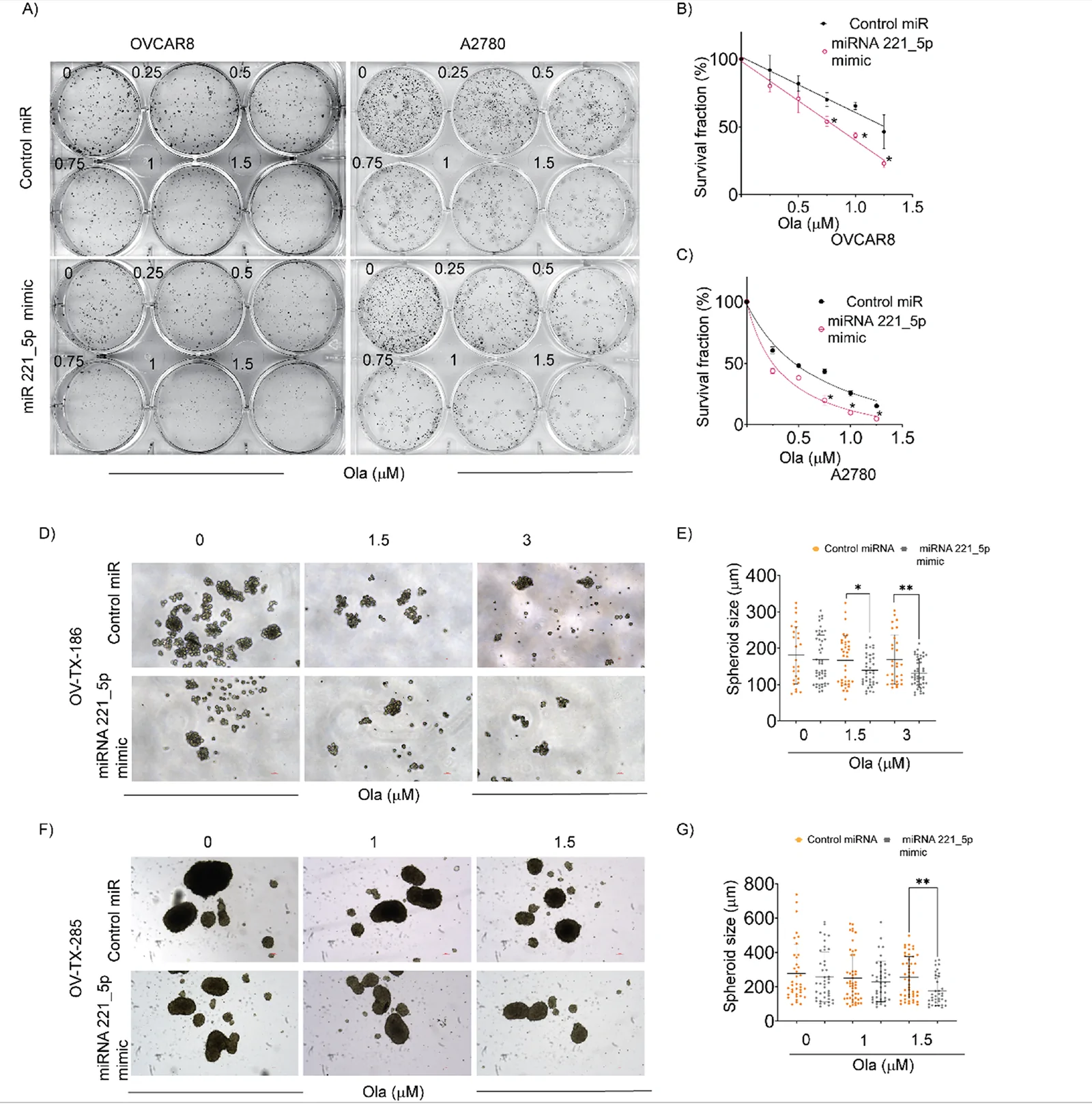
miR-221-5p restoration induces synthetic lethality with olaparib in EOC cell lines and patient-derived organoids.

## Data Availability

The datasets supporting the conclusions of this article is (are) included within the article or its additional files.
